# Changes in Perceived Tinnitus Sound Qualities Following Internet-Based Cognitive Behavioral Therapy for Tinnitus

**DOI:** 10.3390/clinpract15040069

**Published:** 2025-03-27

**Authors:** Vinaya Manchaiah, Gerhard Andersson, Eldré W. Beukes, Marc A. Fagelson, De Wet Swanepoel, David Maidment

**Affiliations:** 1 Department of Otolaryngology-Head and Neck Surgery, University of Colorado School of Medicine, Aurora, CO 80045, USA; dewet.swanepoel@up.ac.za; 2 UCHealth Hearing and Balance, University of Colorado Hospital, Aurora, CO 80045, USA; 3 Virtual Hearing Lab, Collaborative Initiative Between University of Colorado School of Medicine and University of Pretoria, Aurora, CO 80045, USA; eldre.beukes@aru.ac.uk; 4 Department of Speech-Language Pathology and Audiology, University of Pretoria, Pretoria 0028, South Africa; 5 Department of Speech and Hearing, School of Allied Health Sciences, Manipal University, Manipal 576104, India; 6 Department of Behavioral Sciences and Learning, Department of Biomedical and Clinical Sciences, Linköping University, 58225 Linköping, Sweden; gerhard.andersson@liu.se; 7 Department of Clinical Neuroscience, Karolinska Institute, 17176 Stockholm, Sweden; 8 Vision and Hearing Sciences Research Group, School of Psychology and Sports Sciences, Anglia Ruskin University, Cambridge CB1 1PT, UK; 9 Department of Audiology and Speech-Language Pathology, East Tennessee State University, Johnson City, TN 37614, USA; fagelson@mail.etsu.edu; 10 Audiologic Rehabilitation Laboratory, Auditory Vestibular Research Enhancement Award Program, Veterans Affairs Medical Center, Mountain Home, TN 37684, USA; 11 School of Sport, Exercise and Health Sciences, Loughborough University, Loughborough LE11 3TU, UK; d.w.maidment@lboro.ac.uk

**Keywords:** tinnitus, tinnitus sensation, outcome measures, psychometric validation, questionnaire

## Abstract

**Background:** To examine the changes in perceived tinnitus sound qualities following internet-based cognitive behavioral therapy (ICBT) for tinnitus. **Method:** This study was embedded within several clinical trials evaluating the efficacy of ICBT and used a quasi-experimental design (N = 152). Participants completed a series of online questionnaires, including measures of tinnitus sound qualities (Tinnitus Qualities and Impact Questionnaire; TQIQ), tinnitus severity (Tinnitus Functional Index; TFI), anxiety (Generalized Anxiety Disorder-7; GAD-7), depression (Patient Health Questionnaire-9; PHQ-9), insomnia (Insomnia Severity Index; ISI), and health-related quality of life (EQ-5D-5L Visual Analog Scale; VAS). Data were analyzed using a range of parametric and non-parametric statistics, and Cohen’s *d* effect sizes were reported. **Results:** There were no significant differences between the intervention and control groups in sociodemographic and clinical variables at baseline except for anxiety and depression symptoms, which were higher in the intervention group. A statistically significant reduction in tinnitus severity, anxiety, depression, and insomnia was noted post-intervention, with small-to-moderate effect sizes. Statistically significant improvements were also noted for the TQIQ (overall and all subscales) following ICBT compared to the no-intervention group (*p* ≤ 0.028), all with small-to-large effect sizes, except for the loud sounds subscale and for participants with a TQIQ < 38 at baseline, or “mild” perceived qualities of tinnitus (*p* ≥ 0.136). A significantly greater proportion of participants in the intervention group had minimum clinically important differences (38%) on the TQIQ compared to the no-intervention group (9%). **Conclusions:** ICBT can lead to changes in the perceived qualities of tinnitus sound in addition to reducing tinnitus severity and other aspects, such as anxiety, depression, and insomnia. While these findings are preliminary, they highlight that tinnitus distress and perception may be related. However, the study has several limitations including a lack of audiological variables and objective measures. For this reason, the study results must be viewed with caution and must be treated as preliminary.

## 1. Introduction

Tinnitus is the perception of sound without an external source [1]. It is broadly classified into subjective tinnitus, where the sound is heard only by the individual, and objective tinnitus, which can be detected by another person, though subjective tinnitus is the most common. While its exact origin and etiology remain unclear, the leading explanatory models include the neurophysiological and cognitive theories, which describe how tinnitus arises and is maintained in the brain [1].

Tinnitus is a highly heterogeneous condition, varying widely in terms of its origins and manifestations [2]. From the cognitive model perspective, it is important to examine the emotional dimension of tinnitus—such as its impact on stress, anxiety, and quality of life—as it highlights the psychological burden and clinical significance. From the neurophysiological perspective, it is important to examine the tinnitus sounds and their associated perceptual impacts as they differ substantially, with variations in pitch, loudness, and the number of sounds heard reported, both between and within individuals. While many people live well with only minor inconveniences, others face severe challenges, such as difficulties with concentration, sleep disturbances, anxiety, and depression [3]. It is also noteworthy that there is significant correlation between tinnitus severity and other psychological effects such as anxiety and depression [4]. To which extent this also applies when it comes to tinnitus qualities is less well documented, and even a quality like perceived loudness does not necessarily lead to increased annoyance. Contrarily, some studies have also documented a relation between sound qualities, such as tinnitus pitch and loudness, with hearing loss [5].

Tinnitus severity or distress is often measured as a construct of interest for management purposes, although individuals with tinnitus often report that the sounds they hear or experience (i.e., type of sound, how loud it is, how often it is) are themselves problematic. However, there is no standardized measure for self-reported tinnitus perception [6]. Clinicians in practice often ask about aspects like constancy, onset, duration, pitch, and sound quality (e.g., ringing, buzzing) during consultations. While behavioral tests, such as tinnitus pitch and loudness matchings, offer some insights [7], they do not capture all perceptual qualities. We have recently developed and validated the Tinnitus Qualities and Impact Questionnaire (TQIQ), which is a measure of perceived qualities of tinnitus sound [8]. The TQIQ focuses on acoustic characteristics, such as pitch or loudness, that show weak links to severity [9], as well as the interaction between tinnitus sounds and its impact.

Qualitative studies on individuals who have undergone psychological therapies such as internet-based cognitive behavioral therapy (ICBT) indicate that some individuals notice changes in tinnitus sound in addition to a perceived reduction of their distress [10]. While no definitive treatment exists based on the current knowledge, it would be valuable to explore if interventions can meaningfully alter tinnitus sounds—such as reducing the number of sounds, as well as their loudness, bandwidth, or frequency. Due to limited standardized measures of tinnitus sounds, these effects remain largely unexplored.

In our recent study, we examined and reported the psychometric properties of the newly developed TQIQ in terms of content and convergent validity, internal consistency reliability, and floor and ceiling effects by demonstrating the acceptable psychometric properties of this self-reported instrument [8]. In this study, we aim to examine potential changes in perceived qualities of tinnitus sound following ICBT using the TQIQ.

## 2. Methods

### 2.1. Study Design

This study employed a quasi-experimental design and was embedded within several clinical trials (ClinicalTrials.gov registration numbers: NCT04004260 and NCT04335812) investigating ICBT for tinnitus [11,12,13]. Ethical approval was obtained from the Institutional Review Board at Lamar University, Beaumont, TX, USA (IRB-FY17-209 approved on 7 June 2019; IRB-FY20-200 approved on 2 April 2020).

### 2.2. Participants

Participants in this study were compiled from larger clinical trials [11,12,13] and had sought help to manage their tinnitus through an eight-week ICBT program. All eligible participants (N = 152) from the trials were included in the present study. Of these, 107 individuals were in the intervention group, and the remaining 45 were in the control group that did not receive ICBT. It is noteworthy that the experimental and control groups were not equal in size.

The participants were adults (18+ years) living in the US, fluent in English, with computer and internet access, and had experienced tinnitus for at least three months. The three-month duration of tinnitus was necessary to ensure only participants with chronic tinnitus were offered intense psychological interventions such as ICBT. Eligibility was not restricted by hearing level or hearing aid use, and participants were included based on their self-identified need for a tinnitus intervention, regardless of tinnitus severity scores. Exclusion criteria included pulsatile, objective, or unilateral tinnitus that had not been medically evaluated, ongoing medical investigations, major medical conditions preventing participation, or current tinnitus therapy during the study.

### 2.3. Intervention

The ICBT is an evidence-based, structured intervention designed to help individuals manage tinnitus-related distress through online modules. This program is developed based on the cognitive behavioral model of tinnitus and is offered over 8-weeks, with minimal guidance by the therapist. ICBT is based on traditional CBT principles, focusing on cognitive restructuring, relaxation techniques, and behavioral strategies to reduce the negative emotional and psychological impact of tinnitus. Users are provided access to several models that are released weekly. These modules have a mixture of text, videos, exercises and quizzes. In addition, users can also contact the therapist if they have any questions through a secured messaging system. Studies have shown that ICBT is effective in improving tinnitus distress, anxiety, depression, and overall quality of life, with outcomes comparable to in-person CBT [14]. Supplementary Table S1 provides more details about the ICBT intervention including the exercises and suggested reading time. The full-details of the ICBT interventions are provided in a textbook [15].

### 2.4. Data Collection

Participants completed online questionnaires throughout the study. All the study participants were instructed to complete all self-reported measures as required by the study. Additionally, the participants completed a set of standardized, validated measures at both baseline and post-intervention (immediately after completing the ICBT intervention for tinnitus), which included the following assessments:

- Tinnitus severity: Tinnitus Functional Index (TFI; [16]);
- Anxiety symptoms: Generalized Anxiety Disorder-7 (GAD-7; [17]);
- Depressive symptoms: Patient Health Questionnaire-9 (PHQ-9; [18]);
- Sleep disturbance: Insomnia Severity Index (ISI; [19]);
- Health-related quality of life (HRQoL): EQ-5D-5L Visual Analog Scale (VAS; [20]).

The newly developed TQIQ provided in Supplementary Table S2 [8] was administered weekly during the 8-week intervention or weekly check-in (waiting) period. For the purpose of this study, the first- and last-week measures were used as the pre-intervention baseline and post-intervention measures, respectively. The TQIQ has 10 items, covering dimensions such as loudness, pitch, complexity, frequency, coexisting, distractability, maskability, mood, loud sounds, and sensitivity. The TQIQ has two sub-scales: (a) internal tinnitus qualities, and (b) external tinnitus qualities. Each item is rated on an 11-point Likert scale (0 to 10), and the total scores can range from 0 to 100. Scores from 0 to 37 suggest a minimal effect on tinnitus qualities, while scores from 38 to 51 suggest a moderate effect, and scores ≥ 52 suggest a severe effect on tinnitus qualities. A pre–post reduction of 19 points is considered as the Minimum Clinical Important Difference (MCID), as defined in the validation study [8].

### 2.5. Data Analysis

The data analyses were performed using the IBM SPSS Statistics software, version 29.0.1.0 (171). Although some measures violated normality, as tested by Shapiro–Wilk/Kolmogorov–Smirnov tests, none violated the assumption of homogeneity of variance (Levene’s test: *p* > 0.05). ANOVA is generally robust to violations of normality when homogeneity of variance is maintained.

The differences between groups (i.e., ICBT versus no intervention) at baseline for demographics (e.g., age, gender, employment status) and outcome measure scores (i.e., tinnitus distress, anxiety, depression, insomnia, HRQoL, tinnitus sound qualities) were examined using Chi-squared tests for categorical data and independent *t*-tests for continuous data.

For each outcome measure (total and subscale scores), univariate generalized linear model (GLM) analyses were performed with group as the fixed factor. Change in score (or change from baseline) was the dependent variable, where a single measurement was created for each participant by subtracting the post-intervention score from the pre-intervention (or baseline) measurement. Effect sizes (ES [21]) and 95% confidence intervals (CIs) for within-group and between-group differences are reported, where ES are classed as small (0.20), moderate (0.50), and large (0.80), and positive or negative signs indicating that the effect increases or decreases the mean, respectively. A Bonferroni correction to account for multiple comparisons was applied for each outcome measure separately, and adjusted *p*-values are reported. Significance was set to *p* < 0.05.

Based on our previous validation of the TQIQ [8], participants were also divided into two sperate categories: (i) overall difference score <19, or (ii) overall difference score ≥ 19 (i.e., MCID). The proportions of participants within each category were compared between ICBT intervention groups using Chi-square.

## 3. Results

### 3.1. Study Population

The demographic characteristics (Table 1) did not statistically differ between the ICBT and no-intervention groups (*p* ≥ 0.098). In addition, as illustrated in Table 2, the mean scores for each outcome measure at baseline did not significantly differ between the ICBT and no-intervention groups following Bonferroni correction for multiple comparisons (*p* ≥ 0.153), with the exception of GAD-7 and PHQ-9, for which significantly higher scores at baseline were reported regarding generalized anxiety (*t*(150) = −2.68, *p* = 0.009) and depression (*t*(150) = −2.38, *p* = 0.019) in the ICBT relative to the no-intervention control group. As no statistically significant differences between experimental and control groups were found, the sample was appropriate for further analyses.

### 3.2. Change from Pre- (Baseline) to Post-Intervention (8 Weeks)

The mean change from pre- to post-intervention and the differences between the ICBT and no-intervention groups for all outcome measures are shown in Table 3. Pre- and post-intervention mean scores are provided in Supplementary Table S2. The TFI overall scores, GAD-7, PHQ-9, and ISI scores all significantly improved in the ICBT group compared to the no-intervention group (*p* ≤ 0.010), all with small-to-moderate effect sizes. For EQ-5D-5L VAS scores, there were no statistically significant differences between groups (*p* ≥ 0.136).

**Figure 1:**
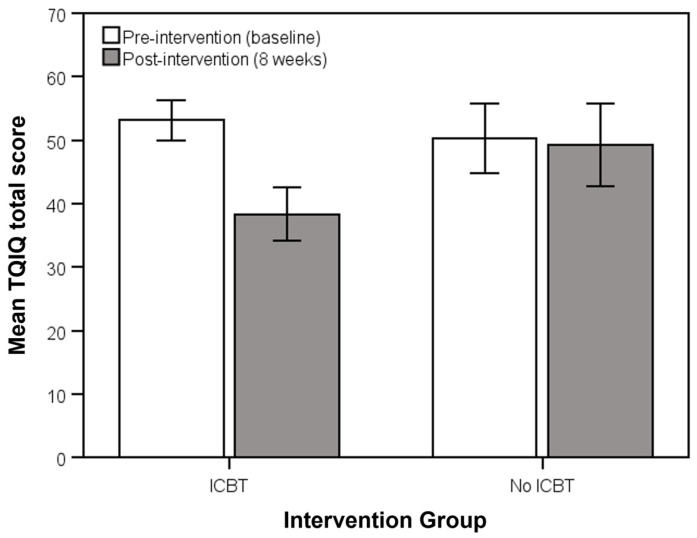
Mean (95% CI) for Tinnitus Qualities and Impact Questionnaire (TQIQ) total scores pre- and post-intervention for participants who did and did not receive internet-based cognitive behavioral therapy (ICBT).

**Figure 2:**
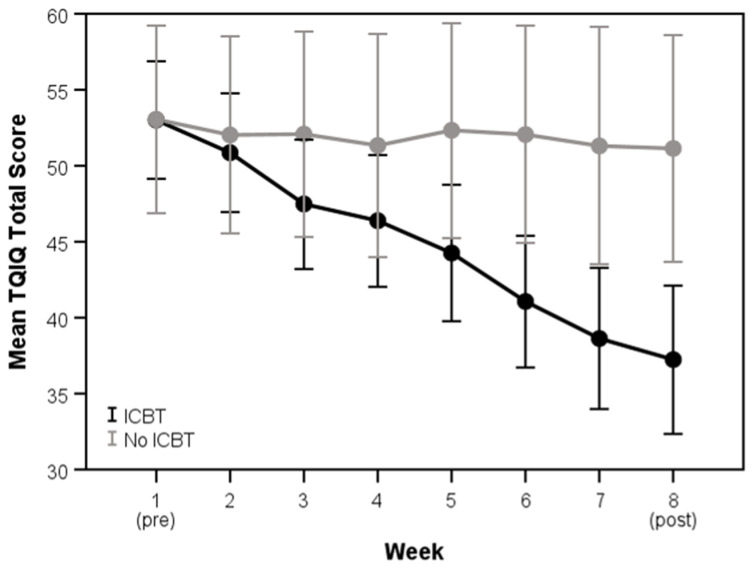
Mean (95% CI) weekly Tinnitus Qualities and Impact Questionnaire (TQIQ) total scores for participants who did and did not receive internet-based cognitive behavioral therapy (ICBT).

For the TQIQ (total and all subscales), scores significantly improved in the ICBT group compared to the no-intervention group (*p* ≤ 0.028), all with small-to-large effect sizes. The only exceptions to this trend were scores on the loud sounds subscale, as well as TQIQ total scores for participants with a TQIQ <38 at baseline, or “mild” perceived qualities of tinnitus (*p* ≥ 0.136). The mean changes in TQIQ total scores before and after the intervention, as well as the weekly TQIQ total scores for the ICBT intervention and no-intervention groups, are also shown in Figure 1 and Figure 2, respectively. A repeated measures ANOVA, with Week as the within-subjects factor and Group as the between-subjects factor, showed a significant Week x Group interaction (*F*(7, 798) = 11.85, *p* < 0.001). Although there were no significant differences between groups in weeks 1 to 5 (*p* ≥ 0.051), mean TQIQ total scores were significantly lower in the ICBT group compared to the no-intervention group in weeks.

### 3.3. TQIQ Minimum Clinical Important Difference (MCID)

Mean TQIQ MCID scores for participants in the ICBT and no-intervention groups with an overall difference score of <19 and ≥19 are shown in Table 4. A significantly greater proportion of participants in the ICBT group had an overall difference score ≥ 19 (38.3%) compared to the no-intervention group (9%), *χ^2^* (1, *N* = 152) = 13.16, *p* < 0.001.

## 4. Discussion

This study provides some preliminary evidence that an ICBT program can significantly alter the perceived sound qualities of tinnitus, as measured by the newly developed TQIQ in individuals with chronic tinnitus. Participants who received the ICBT showed notable improvements in tinnitus sound qualities, including reductions in perceived loudness, pitch, and complexity, compared to the control group. These changes were proportional to reductions in overall tinnitus severity, as indicated by moderate-to-large effect sizes. The findings suggest that ICBT not only reduces tinnitus distress but also positively modifies the sensory experience of tinnitus itself, highlighting its broader impact on the perception of tinnitus symptoms.

### 4.1. Change in Tinnitus Sound Qualities Following ICBT Intervention

Some previous studies have reported strong correlations between tinnitus severity and perceived tinnitus sound qualities such as loudness [5]. The current study demonstrated that an ICBT not only reduces tinnitus distress but also significantly alters the perceived sound qualities of tinnitus. These changes were found to be proportional to reductions in tinnitus severity, as reflected by moderate-to-large effect sizes. Weekly assessments using the TQIQ revealed a pattern of change similar to the reduction in tinnitus severity scores observed in previous ICBT studies [13,22]. Unlike behavioral laboratory measures, which often show weak correlations between tinnitus loudness and severity [23], the TQIQ, a self-reported instrument, demonstrated a strong relationship with tinnitus distress, aligning with findings from its validation study [8].

Two mechanisms could explain these findings. First, ICBT for tinnitus has significant research evidence supporting its role in reducing tinnitus distress, as shown in numerous controlled studies [14] with medium-to-large effect sizes. While studies documenting the objective neurophysiological changes (i.e., amplitude of N1 and P3 in cortical auditory-evoked potentials) following intervention in tinnitus are sparse [24], it is likely that these interventions indeed change the tinnitus sound, as it is maintained by central mechanisms. Second, by equipping individuals with cognitive and behavioral strategies to shift their focus away from tinnitus, ICBT may reduce the salience and awareness of tinnitus sounds, even if they persist. However, further research is needed to disentangle the relative contributions of these psychological and neurophysiological mechanisms. Moreover, there are a range of tinnitus therapies, such as tinnitus educational counseling, Tinnitus Retraining Therapy (TRT) and tinnitus masking, which have good research evidence supporting their roles in tinnitus severity. Further work is needed to examine what changes in tinnitus sound qualities occur following such interventions and their relation to tinnitus severity.

### 4.2. Importance of Measuring Tinnitus Sound Qualities

Despite the availability of various management approaches, there exists no definitive treatment for tinnitus [25]. However, anecdotal reports in the clinics and through social media indicate that many individuals with tinnitus continue to seek out options that may silence or cure their tinnitus [26]. One reasonable option for individuals who are looking for such options is to undergo interventions that could change the tinnitus sound qualities in a meaningful way. This may include reducing the loudness, hearing fewer sounds, and hearing tinnitus less frequently. However, due to a lack of validated and standardized measurement instruments, such interventions have not been measured and reported well in the tinnitus literature [6]. The TQIQ addresses this gap by providing a standardized, patient-reported measure that captures meaningful changes in tinnitus sound qualities. As demonstrated in the current study, the TQIQ can serve as a valuable supplementary measure alongside traditional assessments of tinnitus distress or severity, potentially guiding personalized treatment strategies and enhancing clinical outcomes.

### 4.3. Study Limitations and Future Directions

The study has several limitations, and the results must be considered in light of these. Firstly, the study was embedded in clinical trials focusing on examining the efficacy of ICBT. While this provided an opportunity to study the effects, the data collection process was set up for the ICBT study, and the burden of completing many other measures may have impacted responses to some degree. Secondly, as the study sample included people seeking psychological help for tinnitus, there is a possible sampling bias. Third, the study does not have some key audiological variables (e.g., hearing loss) for the participations, making the results less generalizable. Fourth, the study sample was unequally distributed between experimental and control groups (with fewer participants in the control group), potentially causing some sampling bias. Finally, while the study shows changes in tinnitus sound qualities in self-reported measures, these changes were not studied in relation to behavioral and objective measures. For this reason, it would be useful to replicate this study in a clinical sample and correlate the results with existing behavioral measures such as tinnitus pitch matching and loudness matching. It would also be useful to study possible changes in tinnitus sound qualities following other interventions such as sound therapy.

## 5. Conclusions

The current study is the first to report a change in perceived tinnitus sound qualities following a psychological intervention. These changes are closely aligned with reductions in tinnitus severity, underscoring the potential of ICBT to not only address the psychological burden but also the sensory characteristics of tinnitus. These preliminary findings support the hypothesis that the acoustic properties of tinnitus are linked to its severity and distress levels. The TQIQ, as a self-reported measure, presents a promising tool for clinicians and researchers, complementing established measures like the TFI and assessments of comorbid conditions such as anxiety and depression.

## Figures and Tables

**Table 1:** Baseline socio-demographic information for participants who did and did not receive internet-based cognitive behavioral therapy (ICBT). Note: SD = standard deviation, Chi-squared tests for categorical data and independent *t*-tests for continuous data for examining association between demographic and study groups.

Socio-Demographic Variable	Overall *N* = 152	ICBT *n* = 107	No Intervention *n* = 45	*p*-Value
Age (years), mean (SD)	57.3 (11.8)	56.5 (12.3)	59.3 (10.3)	0.179
Gender (n)				
Male	59 (38.8%)	37 (34.6%)	22 (48.9%)	0.098
Female	93 (61.2%)	70 (65.4%)	23 (51.1%)	
Employment status (n)				
Entry level/unskilled work	2 (1.3%)	2 (1.9%)	0 (0.0%)	0.141
Skilled/professional work	85 (55.9%)	64 (59.8%)	21 (46.7%)	
Retired	54 (35.5%)	36 (33.6%)	18 (40.0%)	
Not working/unemployed	11 (7.2%)	5 (4.7%)	6 (13.3%)	
Education (n)				
<High School	1 (0.7)	1 (0.9%)	0 (0.0%)	0.110
High School	16 (10.5)	10 (9.3%)	6 (13.3%)	
Some college but not degree	43 (28.3)	25 (23.4%)	18 (40.0%)	
>University degree	92 (60.5)	71 (66.4%)	21 (46.7%)	
Tinnitus duration (years), mean (SD)	13.2 (14.9)	12.8 (15.9)	14.0 (12.0)	0.293
Frequency of tinnitus awareness, mean (SD)				
Morning	2.6 (0.7)	2.6 (0.6)	2.6 (0.7)	0.949
Afternoon	2.3 (0.7)	2.2 (0.7)	2.5 (0.7)	0.064
Evening	2.6 (0.6)	2.6 (0.6)	2.7 (0.5)	0.219
Night	2.3 (0.9)	2.2 (0.9)	2.4 (0.8)	0.322

**Table 2:** Mean (standard deviation) scores for each outcome measure at baseline for participants who did and did not receive internet-based cognitive behavioral therapy (ICBT) intervention. Note: TFI = Tinnitus Functional Index, GAD = Generalized Anxiety Disorder, PHQ = Patient Health Questionnaire, ISI = Insomnia Severity Index, TQIQ = Tinnitus Qualities and Impact Questionnaire. Independent *t*-tests were used to examine associations between demographic and study groups.

Outcome Measures	Overall *N* = 152	ICBT *n* = 107	No Intervention *n* = 45	*p*-Value	Adjusted *p*-Value
TFI Total	54.54 (21.69)	54.74 (22.19)	54.07 (19.12)	0.860	1.00
GAD-7	6.97 (5.57)	7.67 (5.81)	5.31 (4.57)	**0.009**	**0.009**
PHQ-9	7.44 (5.85)	8.09 (6.17)	5.89 (4.74)	**0.019**	**0.019**
ISI	11.31 (6.34)	11.85 (6.42)	10.02 (6.02)	0.105	0.105
EQ-5D-5L VAS	75.80 (15.74)	75.55 (15.91)	76.40 (15.49)	0.763	0.763
TQIQ Total	52.29 (17.33)	53.12 (16.89)	50.31 (18.37)	0.363	1.00
TQIQ Loudness	7.20 (2.20)	7.15 (2.18)	7.33 (2.27)	0.639	1.00
TQIQ Pitch	6.95 (2.65)	6.90 (2.67)	7.09 (2.64)	0.685	1.00
TQIQ Complexity	1.78 (2.40)	1.80 (2.43)	1.73 (2.34)	0.869	1.00
TQIQ Frequency	7.13 (2.39)	7.04 (2.67)	7.33 (2.67)	0.517	1.00
TQIQ Coexisting	5.39 (2.59)	5.67 (2.61)	4.73 (2.43)	**0.041**	0.451
TQIQ Distractibility	4.09 (2.80)	4.06 (2.75)	4.16 (2.94)	0.842	1.00
TQIQ Maskability	4.66 (2.80)	4.64 (2.74)	4.71 (2.97)	0.880	1.00
TQIQ Mood	3.98 (3.13)	4.38 (3.05)	3.02 (3.12)	**0.014**	0.154
TQIQ Loud sounds	5.41 (3.38)	5.50 (3.34)	5.18 (3.49)	0.588	1.00
TQIQ Sensitivity	5.70 (2.88)	5.98 (2.71)	5.02 (3.17)	0.060	0.660
TQIQ Internal qualities *	5.90 (2.08)	5.91 (2.06)	5.89 (2.14)	0.967	1.00
TQIQ External qualities **	5.03 (2.42)	5.29 (2.34)	4.41 (2.53)	0.040	0.080
TQIQ Mild (score 0–37)	26.08 (7.52)	25.53 (6.60)	26.82 (8.91)	0.676	1.00
TQIQ Significant (score 38–51)	44.90 (3.60)	44.92 (3.52)	44.85 (3.95)	0.953	1.00
TQIQ Severe (score ≥ 52)	65.84 (10.64)	65.79 (10.76)	66.00 (10.58)	0.938	1.00

Bold indicates significant (*p* < 0.05) difference between intervention and no intervention. * Mean score derived from six TQIQ items: frequency, distractibility, maskability, pitch, loudness, coexisting). ** Mean score derived from three TQIQ items: loud sounds, sensitivity, mood.

**Table 3:** Mean change from pre- (baseline) to post-intervention (8 weeks) and differences between intervention and no-intervention groups for all outcome measures. Note: TFI = Tinnitus Functional Index, GAD= Generalized Anxiety Disorder, PHQ = Patient Health Questionnaire, ISI = Insomnia Severity Index, TQIQ = Tinnitus Qualities and Impact Questionnaire, Mean ∆ = mean change, CI = Confidence Interval, ES = Effect size (Cohen’s d). Univariate generalized linear model (GLM) analyses were performed with group as the fixed factor. Cohens’ d effect sizes are classed as small (0.20), moderate (0.50), and large (0.80).

Outcome Measures	ICBT Mean ∆ (95% CI)	No Intervention Mean ∆ (95% CI)	Difference Between Groups		
			Mean ∆ (95% CI)	ES	*p*-Value
TFI Total	26.03 (21.83,30.23)	15.79 (10.06,21.53)	10.24 (−17.99,−2.49)	0.50	**0.010**
GAD-7	3.59 (2.63,4.55)	0.97 (−0.27,2.21)	2.62 (0.86,4.38)	0.55	**0.002**
PHQ-9	4.04 (2.92,5.16)	1.56 (0.15,2.96)	2.48 (0.45,4.52)	0.47	**0.007**
ISI	4.91 (93.82,6.00)	2.28 (0.95,3.61)	2.63 (0.65,4.61)	0.51	**0.003**
EQ-5D-5L VAS	−4.26 (−6.84,−1.67)	−0.75 (−3.58,2.08)	−3.51 (−8.13,1.12)	−0.29	0.136
TQIQ Total	14.82 (11.83,17.82)	1.04 (−2.31,4.40)	13.78 (8.70,18.86)	0.95	**<0.001**
TQIQ Loudness	2.05 (1.60,2.49)	0.66 (0.11,1.23)	1.38 (0.61,2.15)	0.63	**<0.001**
TQIQ Pitch	2.53 (2.04,3.00)	0.93 (0.43,1.44)	1.59 (0.79,2.39)	0.69	**<0.001**
TQIQ Complexity	0.49 (0.22,0.77)	−0.14 (−0.67,0.40)	0.63 (0.09,1.17)	0.41	**0.024**
TQIQ Frequency	2.10 (1.62,2.56)	0.20 (−0.28,0.68)	1.89 (1.11,2.68)	0.85	**<0.001**
TQIQ Coexisting	1.69 (1.25,2.14)	0.38 (−0.17,0.93)	1.31 (0.55,2.08)	0.60	**<0.001**
TQIQ Distractibility	0.96 (0.52,1.40)	−0.76 (−1.36,−0.15)	1.72 (0.94,2.50)	0.77	**<0.001**
TQIQ Maskability	1.22 (0.78,1.67)	−0.64 (−1.29,0.00)	1.87 (1.07,2.67)	0.82	**<0.001**
TQIQ Mood	1.04 (0.46,1.61)	−0.56 (−1.34,0.23)	1.59 (0.58,2.61)	0.55	**0.002**
TQIQ Loud sounds	1.49 (0.94,2.03)	0.73 (−0.10,1.56)	0.75 (−0.24,1.75)	0.27	0.136
TQIQ Sensitivity	1.26 (0.75,1.77)	0.22 (−0.55,1.00)	1.04 (0.12,1.96)	0.40	**0.028**
TQIQ Internal qualities *	1.76 (1.41,2.11)	0.13 (−0.22,0.48)	1.63 (1.14,2.12)	0.98	**<0.001**
TQIQ External qualities **	1.26 (0.86,1.67)	0.13 (−0.44,0.71)	1.13 (0.41,1.85)	0.55	**0.002**
TQIQ Mild (score 0–37)	8.67 (1.75,15.58)	2.27 (−5.16,9.71)	6.39 (−3.37,16.15)	0.54	0.189
TQIQ Significant (score 38–51)	15.00 (10.37,19.63)	−1.92 (−9.52,5.67)	16.92 (8.20,25.64)	1.26	**<0.001**
TQIQ Severe (score ≥52)	16.36 (11.72,21.00)	2.24 (−2.55,7.03)	14.12 (7.60,20.64)	0.89	**<0.001**

Bold indicates significant (*p* < 0.05) mean change differences between intervention and no-intervention groups. * Mean score derived from six TQIQ items: frequency, distractibility, maskability, pitch, loudness, coexisting. ** Mean score derived from three TQIQ items: loud sounds, sensitivity, mood.

**Table 4:** Tinnitus Qualities and Impact Questionnaire (TQIQ) Minimum Clinical Important Difference (MCID) scores for participants who did and did not receive the internet-based cognitive behavioral therapy (ICBT) intervention. Note: SD = Standard deviation, CI = Confidence Interval. Both upper and lower 95% CI ranges are presented.

TQIQ MCID Category	ICBT			No Intervention		
	N	Mean Diff. (SD)	95% CI	N	Mean Diff. (SD)	95% CI
Overall difference score < 19	66 (61.7%)	4.94 (9.08)	2.71, 7.17	41 (91%)	−0.95 (9.55)	−3.97, 2.06
Overall difference score ≥ 19	41 (38.3%)	30.73 (9.67)	27.68, 33.78	4 (9%)	21.50 (1.73)	18.74, 24.26

## Data Availability

The data that support the findings of this study are openly available in Figshare at http://doi.org/10.6084/m9.figshare.13681924 (accessed on 5 November 2024).

## Supplementary Materials

The following supporting information can be downloaded at https://www.mdpi.com/article/10.3390/clinpract15040069/s1: Table S1: The comprehensive nature of the ICBT intervention offered (Note: The order of the modules is designed to use prior learning to later modules, e.g., need to master deep relaxation before being able to do quick relaxation); Table S2: The Tinnitus Qualities and Impact Questionnaire (TQIQ); Table S3: Mean (standard deviation) overall and subscale scores for each outcome measure pre- (baseline) and post-intervention (8 weeks).
