# Supplementary material for: Changes in Perceived Tinnitus Sound Qualities Following Internet-Based Cognitive Behavioral Therapy for Tinnitus
Source: Clin Pract. 2025 Mar 27;15(4):69. doi: 10.3390/clinpract15040069 (PMC12025759; doi:10.3390/clinpract15040069)
Supplement: Supplementary file 1 [file clinpract-15-00069-s001.zip › clinpract-3507685-supplementary.pdf]

Table S1. The comprehensive nature of the ICBT intervention offered (Note: The order of the modules is designed to use prior learning to later modules, e.g. need to master deep relaxation before being able to do quick relaxation).

Modules	Week	Content	Video	Short worksheets or quizzes	Intervention load	
					Reading time	Daily practicing
1	1	Program rationale and outline	1	3	15 mins	Setting goals
2	1	Tinnitus overview	1	4	15 mins	Reading the module
3	1	Deep relaxation	2	3	15 mins	Twice a day for 10-15 minutes
4	2	Positive imagery	1	5	10 mins	Twice a day for 5minutes
5	2	Deep breathing	1	5	10 mins	Twice a day for 5 minutes
6	3	Changing views	0	7	10 mins	Once a day for 5 minutes
7	3	Entire body relaxation	1	3	10 mins	Twice a day for 5 minutes
18	3	Sound enrichment*	1	2	10 mins	As required
8	4	Shifting focus	0	3	10 mins	4 times a day for 2 minutes
9	4	Frequent relaxation	1	3	10 mins	5-10 times 1-2 minutes
19	4	Sleep guidelines*	1	7	15 mins	Implement daily
10	5	Thinking patterns	0	4	15 mins	3 times a week for 10 minutes
11	5	Quick relaxation	1	3	10 mins	7-15 times a day for up to 1 minute
20	5	Improving focus*	1	2	10 mins	As required
12	6	Challenging thoughts	1	3	15 mins	4 times a week for 5 minutes
13	6	Relaxation routine	0	2	10 mins	Deep relaxation twice a week,<br>Frequent relation 8 times a day, Rapid relaxation during, before or after difficult situations
21	6	Sound tolerance*	1	4	15 mins	As required, 1-2 minutes and increasing
14	7	Being mindful	1	2	10 mins	2-5 times a day during normal activities
22	7	Listening tips*	1	2	15 mins	As required
15	8	Listening to tinnitus	0	3	10 mins	Once a day
16	8	Key point summary	0	0	15 mins	Reading the module
17	8	Future planning	0	4	15 mins	Future plan

Note: *=optional modules

Table S2. The Tinnitus Qualities and Impact Questionnaire (TQIQ)
Instructions: Please respond to the following questions. Select only one of the numbers from 0 to 10 for each question. Select a lower score (0–3) when an aspect has not been a problem. Select a higher number (7–10) when an aspect has been a big problem. If it is only a moderate problem, select a middle number (4–6).

Domain	Question	Select one number for each question.										
Loudness	1. How loud has your tinnitus been?	0 = Hardly noticeable	1	2	3	4	5	6	7	8	9	10 = Very loud
Pitch	2. How annoyed are you with the pitch (or tone) of your tinnitus?	0 = Not at all	1	2	3	4	5	6	7	8	9	10 = Very annoyed
Complexity	3. How many different types of sound do you hear?	0 = A single sound	1	2	3	4	5	6	7	8	9	10 = More sounds than I can count (10+)
Frequency	4. How often are you aware of your tinnitus?	0 = Rarely aware	1	2	3	4	5	6	7	8	9	10 = Always aware
Coexisting	5. How easily have you lived with having tinnitus?	0 = Very easily	1	2	3	4	5	6	7	8	9	10 = Really struggling
Distractibility	6. How much do you notice your tinnitus when you are busy doing other things?	0 = Hardly noticeable	1	2	3	4	5	6	7	8	9	10 = Notice it a lot
Maskability	7. How much do you notice your tinnitus when there are other sounds around you?	0 = Hardly notice it	1	2	3	4	5	6	7	8	9	10 = Notice it a lot
Mood	8. How much does your mood affect your tinnitus?	0 = Not at all	1	2	3	4	5	6	7	8	9	10 = Very much
Loud sounds	9. How has hearing loud noise affected your tinnitus?	0 = Not at all	1	2	3	4	5	6	7	8	9	10 = Very much
Sensitivity	10. How sensitive are you to sounds you hear around you?	0 = Not at all	1	2	3	4	5	6	7	8	9	10 = Very sensitive
	**How to score?**<br>Add all the scores for questions 1 through 10. Add the weekly score to Table 28-3. Lower scores indicate fewer problems. These scores should decrease over time.											

Frequency of Tinnitus Awareness Questionnaire

Please rate how aware you are of your tinnitus. Select only one of the numbers between 0 and 4 for each question. Select lower scores (0–2) when you are not very aware of your tinnitus. Select higher numbers (3–4) when you are frequently aware of your tinnitus.

	**0 = Never aware**	**1 = Seldom aware**	**2 = Sometimes aware**	**3 = Often aware**	**4 = Always aware**
1. In the morning					
2. In the afternoon					
3. In the evening					
4. During the night					
**How to score?**<br>Add all the scores for questions 1 through 4. Lower scores indicate fewer problems. These scores should decrease over time.					

Scoring: The scores are calculated by adding responses of the 10 items in the main scale. The scores can range from 0 to 100. Scores from 0-37 suggest a minimal effect on tinnitus qualities, 38-51 suggest a moderate effect, and scores ≥52 suggest a severe effect on tinnitus qualities. A pre-post reduction of 19 points is considered as a minimum clinically meaningful change.

Table S3. Mean (standard deviation) overall and subscale scores for each outcome measure pre- (baseline) and post-intervention (8 weeks).

Outcome Measure	Intervention			No intervention		
	Pre	Post	Change (pre – post)	Pre	Post	Change (pre – post)
TFI Total	54.98 (22.27)	28.95 (22.56)	26.03 (21.48)	53.91 (20.05)	38.12 (22.82)	15.79 (19.19)
GAD-7	7.74 (5.74)	4.13 (4.22)	3.61 (4.84)	4.95 (4.39)	3.87 (3.80)	1.08 (3.67)
PHQ-9	8.23 (6.16)	4.19 (4.53)	4.04 (5.66)	5.81 (5.09)	4.25 (3.86)	1.56 (4.16)
ISI	11.99 (6.55)	7.08 (5.90)	4.91 (5.52)	9.89 (6.44)	7.61 (5.77)	2.28 (3.93)
EQ-5D-5L VAS	75.59 (16.26)	79.85 (13.53)	-4.26 (13.09)	77.64 (14.15)	78.39 (13.89)	-0.75 (8.35)
TQIQ Total	53.12 (16.89)	38.30 (21.81)	14.82 (15.64)	50.31 (18.37)	49.27 (21.62)	1.04 (11.18)
TQIQ Loudness	7.15 (2.18)	5.10 (2.81)	2.05 (2.33)	7.33 (2.27)	6.67 (2.55)	0.66 (1.86)
TQIQ Pitch	6.90 (2.67)	4.37 (2.79)	2.53 (2.50)	7.09 (2.64)	6.16 (3.07)	0.93 (1.67)
TQIQ Complexity	1.80 (2.43)	1.31 (2.02)	0.49 (1.44)	1.73 (2.34)	1.87 (1.96)	-0.14 (1.79)
TQIQ Frequency	7.04 (2.67)	4.94 (2.79)	2.10 (2.46)	7.33 (2.67)	7.13 (2.58)	0.20 (1.59)
TQIQ Co-existing	5.67 (2.61)	3.98 (2.74)	1.69 (2.32)	4.73 (2.43)	4.36 (2.76)	0.38 (1.84)
TQIQ Distractibility	4.06 (2.75)	3.09 (2.66)	0.96 (2.31)	4.16 (2.94)	4.91 (2.93)	-0.76 (2.01)
TQIQ Maskability	4.64 (2.74)	3.41 (2.72)	1.22 (2.33)	4.71 (2.97)	5.36 (3.07)	-0.64 (2.15)
TQIQ Mood	4.38 (3.05)	3.35 (3.06)	1.04 (3.01)	3.02 (3.12)	3.58 (2.09)	-0.56 (2.62)
TQIQ Loud sounds	5.50 (3.34)	4.02 (3.40)	1.49 (2.86)	5.18 (3.49)	4.44 (3.57)	0.73 (2.76)
TQIQ Sensitivity	5.98 (2.71)	4.72 (3.30)	1.26 (2.65)	5.02 (3.17)	4.80 (3.30)	0.22 (2.58)
TQIQ Internal qualities*	5.91 (2.06)	4.15 (2.47)	1.76 (1.84)	5.89 (2.14)	5.76 (2.45)	0.13 (1.16)
TQIQ External qualities**	5.29 (2.34)	4.03 (2.82)	1.26 (2.10)	4.41 (2.53)	4.27 (3.09)	0.13 (1.91)
TQIQ Mild (score 0-37)	25.53 (6.60)	16.87 (13.05)	8.67 (12.48)	26.82 (8.91)	24.55 (14.88)	2.27 (11.06)
TQIQ Significant (score 38-51)	44.92 (3.52)	29.92 (13.79)	15.00 (13.67)	44.85 (3.96)	46.80 (13.31)	-1.92 (12.57)
TQIQ Severe (score ≥52)	65.79 (10.76)	49.43 (21.34)	16.36 (17.32)	66.00 (10.58)	63.76 (15.84)	2.24 (10.52)
